# GAKG-RGEKG an Epitope That Provokes Immune Cross-Reactivity between* Prevotella *sp. and Human Collagen: Evidence of Molecular Mimicry in Chronic Periodontitis

**DOI:** 10.1155/2016/5472320

**Published:** 2016-12-25

**Authors:** Gustavo Alberto Obando-Pereda

**Affiliations:** Departamento de Periodoncia, Facultad de Odontología, Universidad Católica de Santa María, Urb. San José s/n Umacollo, Arequipa, Peru

## Abstract

Periodontal disease afflicts 20% of world population. This process usually occurs in the form of being lethargic and chronic, and consequently this disease is known as chronic process. All chronic diseases constantly cause activation of the immune system, and therefore the presentation of microbial peptides which are presented to lymphocytes by professional antigen presenting cells can present microbial peptides very similar to important structures of human economy causing autoimmune diseases, process known as molecular mimicry. Thus, the aim of this study was to verify the presence of molecular mimicry phenomenon between periodontopathogens and human proteins. Blasting microbes of Socransky periodontal complexes against human collagen were performed and then the proteins with similarities were modelled and were screened in the MHI binding virtual methods. The epitopes selected were produced and plasma of chronic periodontal volunteers was obtained and a dot immunobinding assay was performed. Hypothetical protein of* Prevotella *sp. and human collagen epitopes with high similarities were positive for dot immunobinding assay. With this result it can be suggested that the mimicry phenomena can occur on periodontal disease.

## 1. Introduction

Since the publication of the subgingival plaque's microbial complexes by Socransky, there has been more knowledge about the microorganisms which are linked directly to the evolution of the periodontal disease; and it has been possible to determine new therapies for its treatment [1]. Nevertheless, Socransky proposes that subjects that suffer chronic periodontitis have* Porphyromonas gingivalis*,* Treponema denticola*, and* Bacteroides forsythia*, belonging to the red complex [1].

These strict anaerobic bacteria have many factors of virulence that activate the immune system in such a way that the immune response causes the destruction of dental support tissues. Another interesting revision of Ximenes describes the complexes of the supragingival microflora, which demonstrates that there are few differences in comparison to the subgingival complexes [2].

The immune response against these microbial complexes has only one similarity: inflammation. As it is known, the subgingival plaque produces inflammation of the gingiva, but without the destruction of the dental support tissue. It is so that once eliminated the cause (that is to say) by removing the dental plaque, this inflammation disappears; but if the inflammation continues due to the dental plaque accumulation, it can cause the activation of the immune system both innate and adaptive, causing the resorption of the dental support. If this process is not stopped by periodontal therapies, it will eventually cause the exfoliation of the dental pieces [3].

The destruction process of dental support tissues is explained by the activation of the innate cells (neutrophils and macrophages) by the use of the TLRs, mainly the TLR4, to recognize the LPS of predominant gram-negative anaerobic microorganisms in the subgingival plaque and is going to promote the transcription of inflammatory cytosine via NF-kB. Thus, this inflammatory cytosine like TNF-*α*, IL-1*β*, and IL-6 once secreted will be the responsible in charge of the production and secretion of RANKL which will form complex with their RANK receiver for the osteoclasts differentiation and survival mechanisms [3–5].

Concomitant to the innate immunology answer, the periodontal bacteria mobilize mechanisms of the adaptive immune response, being that the professional cells (macrophages and dendritic cells) of the innate immunity, at the moment of recognizing the microorganisms by means of the TLRs, and once engulfed, have the first stimulus for their maturation, and a second cytosine profile will determine the subsequent polarization and activation of the specific antigen lymphocytes via MHC II [6]. The subpopulation of lymphocytes T-helper CD4+ that will predominate against a stimulus will obey the type of present cytosine in the injury site Thus, Th1 lymphocytes will have an associated phenotype to a cellular and proinflammatory answer, being that the Th2 lymphocytes will have an associated phenotype to a humoral and anti-inflammatory answer [7, 8].

On the other hand, the presence of Th17 lymphocytes and the secretion of its cytosine IL-17 will increase the action of the cells of the innate immunity in response to the microorganisms. The rough nature of this answer is going to produce the destruction of the bone support. Nevertheless, the IL-17 also has been found in destroyed tissues by autoimmune diseases, like rheumatoid arthritis. Nevertheless, several authors suppose that when this cytosine appears in the injury, the destruction of the dental support can be maintained, by more periodontal therapies the guest receives, causing an autoimmune periodontitis [3–5].

Many studies relate autoimmune diseases like rheumatoid arthritis, coronary syndrome, and even erythematous lupus with the presence of periodontitis; this can prove that the phenomenon of molecular mimicry can take place in the oral cavity, being in the activation of the immune system able to destroy own tissues [9–12]. The molecular mimicry is defined as the sequences of proteins or similar peptides between a microorganism and tissues of another organism, in this case, humans. This phenomenon is frequently associated with autoimmune diseases [13].

The immunological tolerance for T and B cells supposes many processes of education in which these cells are eliminated if they do not recognize in the first place the histocompatibility major complexes I and II and if they recognize own structures. Consequently, the molecular mimicry does not obey this process of education. It simply happens because some microorganism structure is similar to some component of the guest, causing an autoimmune disease. An example of this process occurs when an immune response against protein m of the* S. pyogenes* causes a crossed answer against cardiac myosin, promoting heart damage [14].

Another example in which this phenomenon occurs is in the pancreatic beta cells, due to the crossed answer by cells T associated with viral infections in diabetes type I [15]. A final example is a similar crossed reaction to the leukotoxin of the* A. actinomycetemcomitans* and to the glycoprotein-B2 being observed during the destruction of the periodontal tissue by the process of molecular mimicry, being that in this study the similarity between the leukotoxin peptide and the glycoprotein-B2 peptide is of 60% [16].

The objective of this study was to verify the presence of the phenomenon of molecular mimicry between periodontal pathogens with human proteins.

## 2. Materials and Methods

### 2.1. Alignment of Sequences: Blast

The free BLAST program (http://blast.ncbi.nlm.nih.gov/Blast.cgi) was employed. First, a general alignment between human proteins versus periodontal pathogens bacteria's proteins described by Socransky was performed. Later, a second individual blast was made by using DNASTAR Lasergene software (Wisconsin, USA) to the proteins that had similarity.

### 2.2. Prediction of Epitope Affinity

The MHCpred free program (http://www.ddg-pharmfac.net/mhcpred/MHCPred/) was used to evaluate the affinity of the found sequence of the* Prevotella *sp. hypothetical protein to be presented by the complex of greater histocompatibility of type II human where we used alleles MHC II HLA-DPA1*∗*01/DPB1*∗*04:01 and HLA-DPA1*∗*01:03/DPB1*∗*02:01.

### 2.3. Protein Modelling

The program in real-time Swiss-Model (http://swissmodel.expasy.org/) was used to accomplish the modelled of proteins. Once obtained, it is tried and it proceeded to accomplish the structural alignment for these proteins with the program PyMOL (https://www.pymol.org/)

### 2.4. Patient Selection and Obtainment of the Dot Immunobinding Technique Assay

Voluntary patients from the Dental Clinic of the Universidad Católica de Santa María with chronic periodontitis were selected. The chronic periodontitis was diagnosed following the periodontal classification of 1999 [17], where there were periodontal pockets of more than 6 mm with accumulation of plaque and alveolar bone loss.

Eleven volunteers were selected and 20 mL of blood was extracted from each patient and then proceeded to obtain the plasma. The plasma obtained was carefully stored for its later use. Afterwards, the peptide in question was obtained from ByoSynthesis (Texas, USA) and later submitted to diva technique, described by Sumi et al. [18].

## 3. Results

### 3.1. Alignment of Sequences: Blast

A microbial peptide could be found with the BLAST method with a similarity of more than 90% to human collagen. This difference only falls to the difference of 1 amino acid for a total epitope of 10 amino acids (Table 1).

### 3.2. Prediction of Epitope Affinity

*Prevotella *sp. follows the alignment for MHC II, with percentiles major to 85% (Boxes 1 and 2).

## 4. Protein Modelling

**Figure 1:**
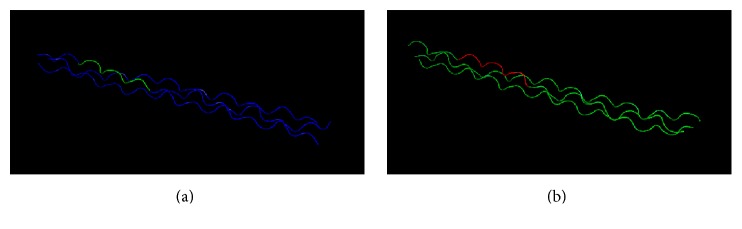
Alfa-1 collagen (a) and* Prevotella *sp., ZP_05918585.1 (b); according to Swiss-Model these proteins present triple-helix configuration.

With the Swiss-Model and PyMOL programs, very similar structures were obtained (Figures 1(a) and 1(b))

These proteins own a structure of 3 helixes of similar characteristics.

## 5. DIBA

**Figure 2:**
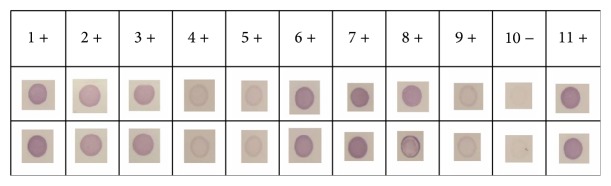
It is observed that in 7 patients there is a strong ligation between the designed bacterial epitope and obtained antibodies of blood plasma.

The results showed a strong positive ligation (++) for 7 of the obtained samples of patients with chronic periodontitis, 3 of them had lighter positive feedback (+) and 1 of them did not obtain ligation (−) (Figure 2).

## 6. Discussion

The periodontal disease, whose etiology is clearly of microbial origin, activates the immune system in such a way that it produces chronic bone resorption leading up to the complete exfoliation of the affected dental pieces, if the aggressive agent is not removed. The activation of the immune system, by consequence, begins with the recognition of the microorganisms by the macrophages with an activation pathway of the TLR2 and TLR4, leading to the apparition of inflammatory cytosines with the consequent expression of RANKL for the osteoclasts activation and survival mechanisms [5, 19]. Nevertheless, in chronic periodontal disease, a constant presence of antigens to lymphocytes takes place due to the contact of microbial biofilm with immune cells; therefore the activation of the adaptive immune system takes place [5, 6, 8, 19].

The* Prevotella* species has been described in the complex orange of Socransky, like bacteria that appear in the beginning stages of the periodontal disease: the gingivitis. This species owns a series of virulence factors (adhesion, competition, horizontal gene transfer, among others) [20] able to produce the appearance of periodontal inflammation. Nevertheless, this specie is not classified like an important periodontal pathogen in this disease.

The molecular mimicry, a theoretical possibility that sequences of epitopes of an organism are similar to some component of another microorganism, proves evident in a periodontal inflammation [16] by the amount of microorganisms present in direct bonding with immune cells. Some studies have been able to corroborate this fact, finding titles of antibodies in injuries of rheumatoid arthritis [16, 21–25], especially against the* P. gingivalis*. In consequence, two scientific articles only corroborate antibodies against* Prevotella *sp. in rheumatoid arthritis [23, 26].

Our results confirm these two findings, besides describing the epitope that generates the molecular mimicry that is not yet described in scientific literature. This epitope by consequence belongs to a protein with unknown function, whose structure seems to be very similar to the human collagen. Besides being reactive for MHC II, the similar bacterial protein similarity with human collagen has been already reported previously and studied [27], which is confirmed by our results. This result gives an important step to explain the origins of rheumatoid arthritis, because antibodies against this bacterial epitope are reactive also, by similarity, against human collagen. The epitope GAKG-RGEKG, in consequence, can be considered as an autoantigen.

Future studies must be directed to discover the protein's function in a matter of* Prevotella *sp. as well as observe this reaction in patients with rheumatoid arthritis.

## 7. Conclusion

With this result the existence of the molecular mimicry phenomenon in the periodontal disease can be suggested, almost in initial stages of the gingival inflammation, giving rise to autoimmune diseases that affect collagen, as a rheumatoid arthritis.

## Figures and Tables

**Box 1:**
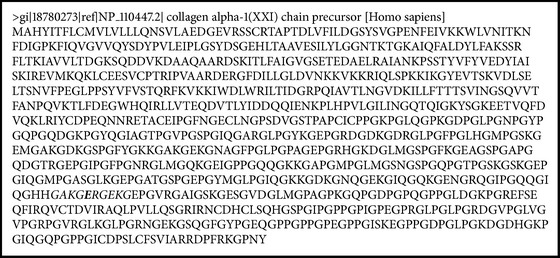
The sequence of aa of the human collagen type 1 protein. The peptide of similar characteristics in human collagen type 1 protein and *Prevotella* sp. protein is observed in italic.

**Box 2:**
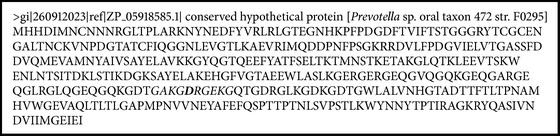
The sequence of aa of the *Prevotella* sp. protein. The peptide of similar characteristics in human collagen type 1 protein and *Prevotella* sp. protein is observed in italic.

**Table 1:** 

PubMed ref	Microorganism	Microbial peptide	Human collagen peptide
ZP_05918585.1	*Prevotella *sp.	GAKG-DRGEKG	GAKG-ERGEKG

Found peptide of 10 aa pertaining to *Prevotella *sp., which owns 9 aa identical to human collagen alpha-1.
